# Some thoughts about what non-mammalian jawed vertebrates are telling us about antigen processing and peptide loading of MHC molecules

**DOI:** 10.1016/j.coi.2022.102218

**Published:** 2022-08

**Authors:** Rebecca Martin, Jim Kaufman

**Affiliations:** 1 Department of Pathology, University of Cambridge, Tennis Court Road, Cambridge CB2 1QP, UK; 2 Department of Veterinary Medicine, University of Cambridge, Madingley Road, Cambridge CB3 0ES, UK; 3 Institute for Immunology and Infection Research, University of Edinburgh, Charlotte Auerbach Road, Edinburgh EH9 3FL, UK

**Keywords:** TAP, transporters associated with antigen presentation, HLA, human leukocyte antigen, BAC, bacterial artificial chromosome, BRD2, bromodomain-containing protein 2, BF-BL, the region including class I (BF) and class II (BLB) genes in the chicken, APLL, antigen processing and peptide loading, XNC, Xenopus nonclassical, CD1, cluster of differentiation antigen 1, MR1, MHC class I-related gene 1, YF, class I gene of the chicken Y region, TAPBPR, TAP-binding protein-related, TAPBPL, TAP-binding protein-like, DO, D-related protein O, YLB, class II B gene of the chicken Y region

## Abstract

The major histocompatibility complex (MHC) of mammals encodes highly polymorphic classical class I and class II molecules with crucial roles in immune responses, as well as various nonclassical molecules encoded by the MHC and elsewhere in the genome that have a variety of functions. These MHC molecules are supported by antigen processing and peptide loading pathways which are well-understood in mammals. This review considers what has been learned about the MHC, MHC molecules and the supporting pathways in non-mammalian jawed vertebrates. From the initial understanding from work with the chicken MHC, a great deal of diversity in the structure and function has been found. Are there underlying principles?

## Highlights

• The genomic organisation of the MHC varies enormously across jawed vertebrates.
• Total numbers of MHC genes vary among vertebrates, with only a few classical MHC genes.
• Some nonclassical MHC and classical pathway genes appear earlier than others.
• Obvious co-evolution within MHC pathways occurs in some species, but not others.
• The promiscuity of interactions may correlate with differences in genomic organisation.

**Current Opinion in Immunology** 2022, **77**:102218

This review comes from a themed issue on **Antigen processing**

Edited by **Andrea Sant** and **Lawrence Stern**

For complete overview of the section, please refer to the article collection, “Antigen Processing (June 2022)”

Available online 7th June 2022

https://doi.org/10.1016/j.coi.2022.102218

0952-7915/© 2022 The Authors. Published by Elsevier Ltd. This is an open access article under the CC BY license (http://creativecommons.org/licenses/by/4.0/).

## Introduction

**Figure 1:**
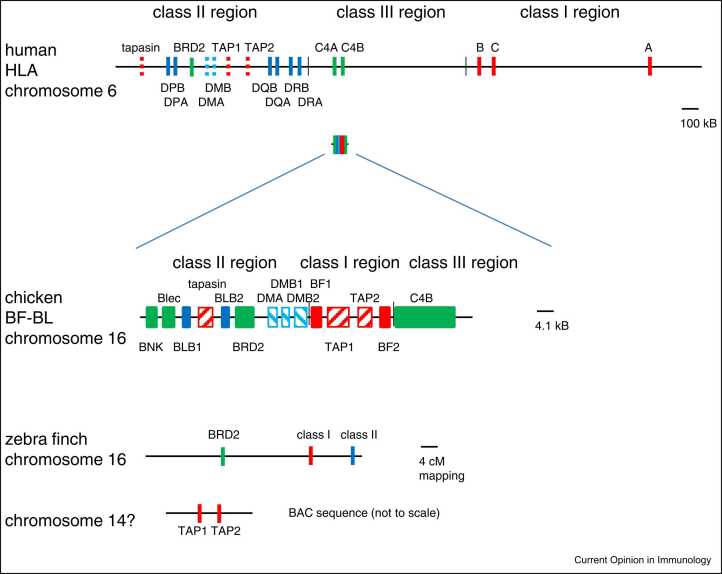
Major differences in MHC organisation between humans, chickens and the passerine bird zebra finch. The top panel shows a textbook representation of the human HLA region on chromosome 6 with the order class II region, class III region and class I region, altogether an enormous region (to scale as indicated) with hundreds of genes not depicted. The middle panel shows the chicken BF-BL region on chromosome 16, first to the same scale as HLA and then magnified to show there very few genes present in the ‘minimal essential MHC’ with the order class II region, class I region and class III region. The bottom panel shows evidence from zebra finch, with the top line indicating the results of a mapping study showing the genetic distances between the BRD2, class I and class II genes, and with the bottom line indicating a BAC containing the TAP1 and TAP2 genes; cytogenetics shows that different microchromosomes bear BRD2-class I-class II genes and TAP genes.

The textbook view of major histocompatibility complex (MHC) organisation is that of humans and mice (Figure 1): overall a huge region with hundreds of genes and riven by recombination, but organised as a class III region with many different kinds of genes, flanked by a class I region with multigene families of classical and nonclassical class I genes amid a variety of framework genes on one side, and by a class II region with a multigene family of class II genes as well as nonpolymorphic antigen processing and peptide loading (APPL) genes on the other side 1, 2. An enormous literature describes the pathways that lead to antigen presentation by classical class I and class II molecules, including the cross-presentation pathways that occur mostly in specialised cells 3, 4.

Top and middle panels modified from [5]; data in bottom panel from 18, 19.

Mammals make up only an estimated 5000 mammalian species out of the roughly 66 000 jawed vertebrate (gnathosome) species with MHC molecules (https://en.wikipedia.org/wiki/Vertebrate). So far, what have we learned about antigen processing, peptide-loading and antigen presentation from the non-mammalian jawed vertebrates (NMJVs)?

For many years, targeted cloning, monoclonal antibodies and genetic mapping provided the only avenues to look at the MHC and MHC molecules outside of mammals, with the most detailed examination for the domestic chicken 5, 6••. More recently, genomic sequencing projects for many NMJVs, the latest by long-read (third generation) technologies 7•, 8•, 9•, have given information about genes and genomic organisation at various levels of resolution and have provided the basis for more detailed studies. Thus far, detailed studies at the protein level have only been reported for chickens.

Given the length of this review, a knowledge of the MHC molecules and APPLs in mammals is assumed, and many seminal reports with original discoveries are not cited since they are covered in later papers or reviews. Statements without citations are supported by references in reviews, including 5, 6••, 10, 11, 12.

## The chicken major histocompatibility complex provided the first model for a major histocompatibility complex of non-mammalian jawed vertebrates

In contrast to typical mammals 5, 6••, 12, the chicken MHC was found to be compact with little evidence for recombination, simple with far fewer genes, and arranged differently, with the class III region on the outside and the polymorphic TAP genes flanked by the two classical class I genes (Figure 1). Only one of the two classical class I genes, BF2, is expressed at a high level, and the peptide-binding specificity of BF2 was found to match the peptide-translocation of the chicken TAPs for each MHC haplotype, suggesting that the lack of frequent recombination allows co-evolution to drive the presence of a single dominantly-expressed class I molecule. A similar situation was suggested for the class II B (BLB) genes and the polymorphic DM genes. In this sense, genomic structure was proposed to determine function, with single dominantly-expressed classical MHC genes leading to strong genetic associations with infectious disease [13]. The presence of single dominantly-expressed classical MHC genes leading to strong genetic associations with infectious disease was also suggested by studies with the bony fish Atlantic salmon [14].

On the basis of the chicken MHC, it was proposed that the ancestral MHC was organised with the class III region on the outside and with the class I and class II genes next to their APPL genes 5, 11. It was envisioned that an inversion occurred on the lineage to placental mammals, with the class III region swinging to the inside, the class I gene(s) swinging to the outside, but with the APPL genes left behind in the region that became the class II region. Recombination between the separated APPL genes and the class I gene(s) meant that the co-evolutionary relationships broke down, so that the APPL genes became average best fits, supplying peptides for whatever class I allele(s) appeared by recombination, thus allowing a multigene family. Moreover, it was suggested that the APPL genes were present in the primordial MHC for co-evolution to set up the pathways. Finally, based on the presence of an NK receptor/ligand pair in the chicken MHC, it was proposed that the original antigen-specific receptors were also present in the primordial MHC, which was the birthplace of the adaptive immune system of jawed vertebrates. Based on different considerations, other researchers came to similar conclusions [15•], but the presence of immune receptors in the primordial MHC has been challenged by recent genome reconstructions [7•].

Just to be clear, it is an idealised version of the chicken MHC that is proposed as a model for the primordial MHC. Among the features that don’t fit are the class II A gene (BLA) located 5 cM away from the BF-BL region, tapasin located in between class II B genes, two CD1 genes located at the edge of the BF-BL region, and variable numbers of nonclassical class I and class II B genes located in the genetically-unlinked Y region on the same chromosome 5, 12. The latest comparison of bird genomes concludes that the ancestral organisation of the avian MHC was compact and simple like the chicken and ratites (the most ancient surviving clade, including ostriches), but without the apparent translocations [16•].

## A wide variety of major histocompatibility complex organisations

Overall, the evidence for MHC organisation remains frustratingly fragmented. Some species, like *Xenopus* frogs, have a very similar organisation to chickens, including a single classical class I gene next to its APLL genes, a class II gene pair next to their APPL genes, a class III region on the outside and even a region of nonclassical class I genes, the XNCs [15•]. The marsupial American opossum has the class III region outside of the class I and class II genes, although the original organisation was missing two class I genes [17]. Birds closely-related to chickens have very similar MHC organisations, and many other birds show a similar organisation with or without some of the translocations [16•].

However, other species differ in a variety of ways, highlighting dynamic changes in the genomic organisation in some lineages. The passerines (perching and song birds which make up roughly 50% of bird species) have dispersed their MHC genes over long distances and even on other chromosomes [16•]. For example, the zebra finch has separated class I and class II genes by 9 cM, and the TAP genes appear to be on a different chromosome 18, 19 (Figure 1). This situation is reminiscent of the marsupial Tammar wallaby, in which the class I genes are dispersed in the telomers of many chromosomes, far away from the TAP genes [20]. In the salamander axolotl, the region containing MHC genes is 100 Mb long, with much apparent duplication [8•].

Some fish, like the cartilaginous sharks (and likely the lobe-finned bony fish, most closely related to the ancestors of tetrapods) have a single MHC, but one or two rounds of genome-wide duplication followed by differential gene silencing have complicated the organisation of the MHC genes in teleosts (the major radiation of ray-finned bony fish). In general, there is a canonical teleost class I region with TAPs, tapasin and inducible proteasome components along with other genes found in the MHC of many jawed vertebrates, with class II and class III region genes spread on different chromosomes [10]. Strikingly, some fish like cod and relatives have lost the class II system altogether, with some of the many class I genes suggested to have taken on class II functions, although there have been concerns 21, 22.

**Figure 2:**
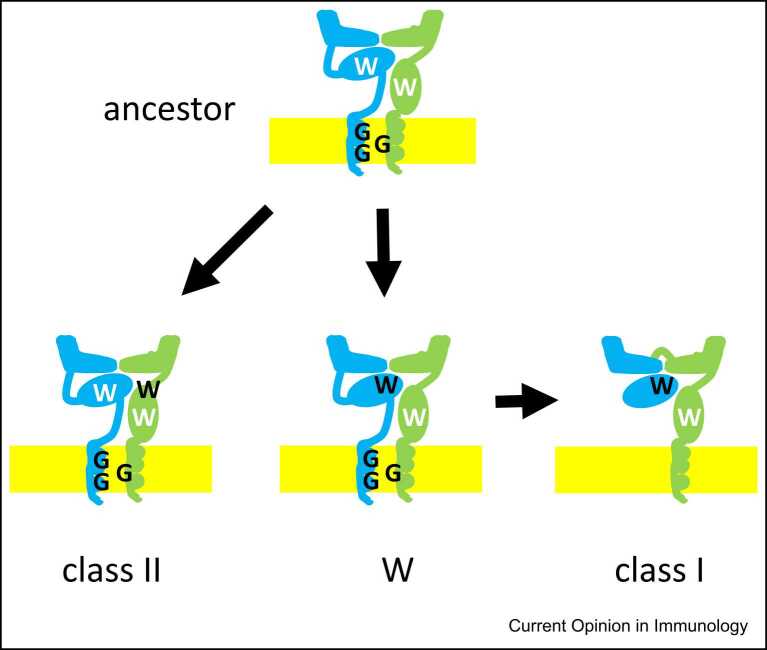
The newly-described W genes show that class II genes preceded class I genes. Two transmembrane chains of roughly equal size constitute the W, class II and proposed ancestral molecules (α chain in blue, β chain in green, membrane in yellow), with the domains rearranged in class I molecules (β_2_-microglobulin in blue, heavy chain in blue and then green). A so-called ‘invariant tryptophan’ (W in white) is found between the β-sheets of most immunoglobulin domains, but is replaced by other hydrophobic residues in the W α2 domain and β_2_-microglobulin. Other changes include the glycines that allow the two transmembrane regions to pack together (G in black), and tryptophans involved in inter-domain interactions (W in black).

For the surviving members of the jawless fish lampreys and hagfish (the earliest fish group to appear in the evolutionary record), there is an adaptive immune system with three kinds of antigen-specific receptors based on leucine-rich repeats, but no genes with obvious similarity to or properties like MHC genes have been reported 23, 24. However, the discovery of the so-called W genes in sharks, various bony fish and salamanders shows that class II genes preceded the appearance of class I genes [25••], finally laying to rest the controversy over which came first [26•]. The W genes are present in AB gene pairs and are expressed as αβ heterodimers with the domain structure of class II molecules, but have many key sequence features of class I molecules. In particular, β_2_-microglobulin and the W β2 domain both lack the so-called invariant tryptophan found in nearly all Ig domains, showing the direction of evolution from class II to W to class I genes (Figure 2). There is no evidence for polymorphism or canonical peptide-binding residues, so the past and present functions of W molecules are unknown 25••, 27.

Figure modified from [27].

### Many major histocompatibility complex genes but only one or a few classical major histocompatibility complex genes

The number of MHC class I and class II genes as well as APPL genes varies considerably among the NMJV, but many species have a single or a dominantly-expressed classical class I gene closely linked to APPL genes, with the remaining class I genes likely to be nonclassical. Classical MHC molecules are defined as those that present peptides to T cells, have high levels of polymorphism and are widely and well-expressed. Nonclassical molecules lack one or more of these features and arise to counter particular threats, appearing and disappearing at different times in evolutionary history, so that some are more ancient and others are present only in particular lineages [28]. As examples, CD1 genes are found in mammals, birds and reptiles, but are thought to have arisen before and been lost from earlier lineages, while MR1 genes are thus far only found in mammals [29]. In mammals, nonclassical genes such as CD1, MR1 and FcRn are found in so-called MHC paralogous regions derived from two rounds of genome-wide duplication at the base of the jawed vertebrates, but their presence has recently been ascribed to translocations [15•].

Among tetrapods, *Xenopus* frogs have a single classical class I gene closely linked to the TAP genes, with two lineages of each (but without detailed sequencing to look for potential co-evolution of peptide translocation and binding specificities), along with many nonclassical XNC genes known to be recognised by natural killer T cells [29]. Only one of the two classical class I (BF) genes in chickens is well-expressed throughout the body, a situation proposed to have been driven by co-evolution with polymorphic APPL genes that are not separated often by recombination. In addition, there are two monomorphic CD1 genes nearby and a variable number of nonclassical YF genes on the same chromosome, all of which bind hydrophobic ligands 12, 30, 31. Ducks also have polymorphic TAPs next to five class I genes, only one of which is a well-expressed classical gene (although another is upregulated upon infection) 32, 33. In contrast, there are many apparently nonclassical class I genes in passerine birds, with the APPL are located at a distance from the classical class I gene(s). As examples, there is one classical class I gene on a separate chromosome from the TAP genes in zebra finch, and only one of the two classical class I genes in sparrows is well-expressed and with the nonpolymorphic TAP genes located far away 18, 34 [Rebecca Martin, PhD thesis, University of Cambridge, 2021]. There are as many as five lineages of class I genes found in fish, but apparently only one classical lineage, and with dramatic expansions of some nonclassical lineages in one or another fish 10, 35, 36. In some bony fish like Atlantic salmon and medaka [10], there are only one or two classical class I genes closely linked to a TAP2 gene, although there are many more nonclassical genes.

There is wide variation in the other APPL genes of the class I pathway. Birds have no inducible proteasome genes, which is surprising since PSMB11 (β5t) is considered essential for positive selection of mammalian thymocytes 37, 38. In contrast, PSMB8 (β8) genes in sharks, bony fish and *Xenopus* frogs have two allelic lineages with different protease specificities, and the inducible proteasome genes in the bony fish zebrafish have copy number variation 39, 40. Tapasin is reported to be absent in ducks, and in fact tapasin family members have been found in only a few passerine bird genomes [32] [Rebecca Martin, PhD thesis, University of Cambridge, 2021]. However, three tapasin family members are found in many other NMJV including chickens: tapasin (TAPBP), TAPBPR and TAPBPL [41]. In birds, only TAP2 has a homologous tapasin-binding site, raising questions of structure and mechanism of the peptide-loading complex compared to mammals [12].

The situation is even less clear for class II and their APPL genes. In a few species, like parrots, axolotls, Atlantic salmon and medaka, there is only a single classical class II gene pair 10, 42, 43, but in most species, there are several classical class II genes described, but without evidence for their relative expression or importance. Some species also have many other (presumably nonclassical) class II genes, such as the zebra finch, Hawaiian honeycreepers, zebrafish and shark 10, 18, 44, 45, 46•. Invariant chain (Ii) genes are found in all NMJV examined, with the exception of those that have lost the entire class II system, such as cod [48]. DM genes are found throughout the tetrapods as well as in lungfish (a lobe-finned fish thought to be most closely related to the ancestors of the tetrapods), but no DM orthologs have been found in other fish 45, 47, 48•. Whether cartilaginous fish and most bony fish have an alternative dedicated chaperone or none at all remains unclear. DO genes have not been found outside of mammals, although the similarity of DO and classical class II genes may mean that some nonclassical class II genes in other species, such as the YLB genes in chickens, could play a similar role.

### Generalists and specialists: a potential explanation for the different major histocompatibility complex strategies

**Figure 3:**
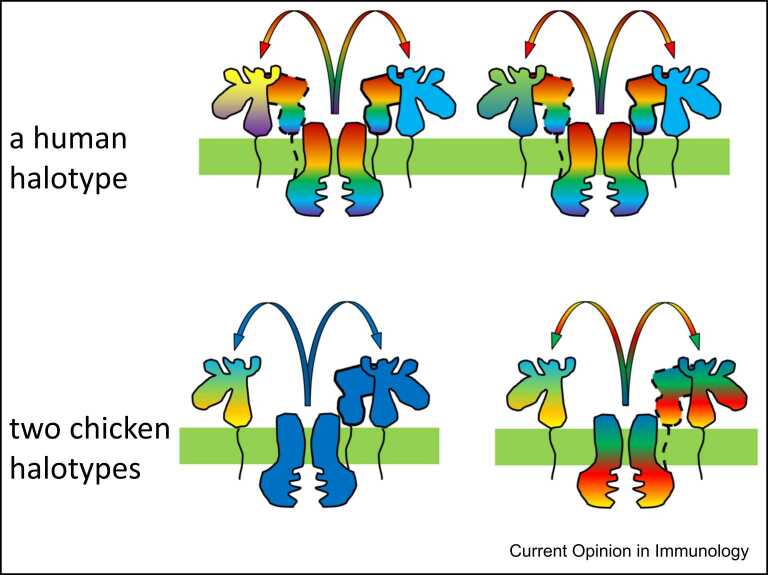
Humans and chickens use combinations of TAP, tapasin and classical class I molecules with different levels of promiscuity to achieve antigen presentation. Above, depicted are the molecules from one human haplotype, with two molecules of a somewhat fastidious class I molecule (reflecting the greater numbers found on the cell surface) and two somewhat promiscuous class I molecules, along with highly promiscuous (and monomorphic) tapasin and TAP molecules. Below, depicted are the molecules from two chicken haplotypes, one with fastidious BF2, tapasin and TAP molecules along with a promiscuous BF1 molecule, and the other with very promiscuous BF2, tapasin and TAP molecules along with a similar promiscuous BF1 molecule (reflecting the lower number of alleles with different peptide-binding sites for BF1 compared to BF2). Solid colours indicate fastidious molecules, rainbows indicate promiscuous molecules with more colours indicating greater promiscuity; thus chicken class I molecules can be more promiscuous than the most promiscuous human class I molecule, while human TAP molecules are much more promiscuous than the most promiscuous chicken TAP molecule. Dotted lines around tapasin indicate relative tapasin-independence of the interaction with promiscuous molecules, while only one tapasin per haplotype is shown for chickens, since only the TAP2 chain has a recognisable binding site for tapasin.

Outside of mammals, natural ligands have been determined only for chicken classical MHC molecules. A suite of properties was found for the dominantly-expressed class I BF2 molecules which varied in a hierarchy, from quite stringent peptide motifs compared to most human class I alleles to extremely promiscuous 5, 6••, 12. The structures showed that the promiscuous BF2 * 2101 molecule remodels the binding site to bind peptides with completely different sequences, while BF2 * 0201 binds peptides with a wide range of hydrophobic anchor residues [49]. Another way to achieve promiscuity arises from one of the highly-conserved residues that bind the C-terminus of the peptide changing from a tyrosine in mammals to an arginine found in all nonmammalian jawed vertebrates (and also in class II molecules), which allows peptides to extend out of the groove at the C-terminus [50]. In terms of peptide loading, the translocation specificity of the TAP correlates with peptide motif of the BF2 molecules in a given MHC haplotype (Figure 3), and there is a haplotype-specific effect on maturation ascribed to tapasin 5, 6••, 12.

In contrast, the BF1 gene is at least ten-fold less well expressed than BF2 and has fewer alleles, many of which have nearly identical peptide-binding motifs 5, 6••, 12. Given the wide range of peptide-translocation specificities among MHC haplotypes, it seems likely that most BF1 alleles are highly promiscuous (Figure 3). What little data exists suggests that BF2 is primarily a ligand for cytotoxic T lymphocytes, while BF1 is primarily a ligand for NK cells; the potential similarities (and differences) between BF1 and HLA-C have not gone unnoticed [51].

A concept of generalist and specialist alleles arose from these findings for BF2 molecules, based on a suite of properties established during peptide loading 5, 6••, 49. Promiscuous alleles bind a wide variety of peptides and protect against a wide variety of common pathogens, but they have relatively low stability and low expression on the cell surface. Fastidious alleles have stringent binding requirements resulting in narrow peptide repertoire, higher stability and higher cell surface expression. Comparison with human HLA-B alleles shows that these features are a fundamental property of some classical class I loci, with the fastidious well-expressed human alleles binding special peptides to resist particular pathogens, including elite controllers of progression from HIV infection to AIDS. Taken together, the promiscuous molecules generally protect from many pathogens, while the fastidious molecules protect against a few special pathogens. In chickens, the peptide-translocation specificity of the TAP molecule correlates with the peptide-binding specificity as well as the peptide repertoire of the BF2 molecule (with tapasin alleles yet to be rigorously tested), while in humans the correlation is with tapasin-dependence (Figure 3), with fastidious alleles requiring tapasin 5, 6••, 12, 52•.

**Figure 4:**
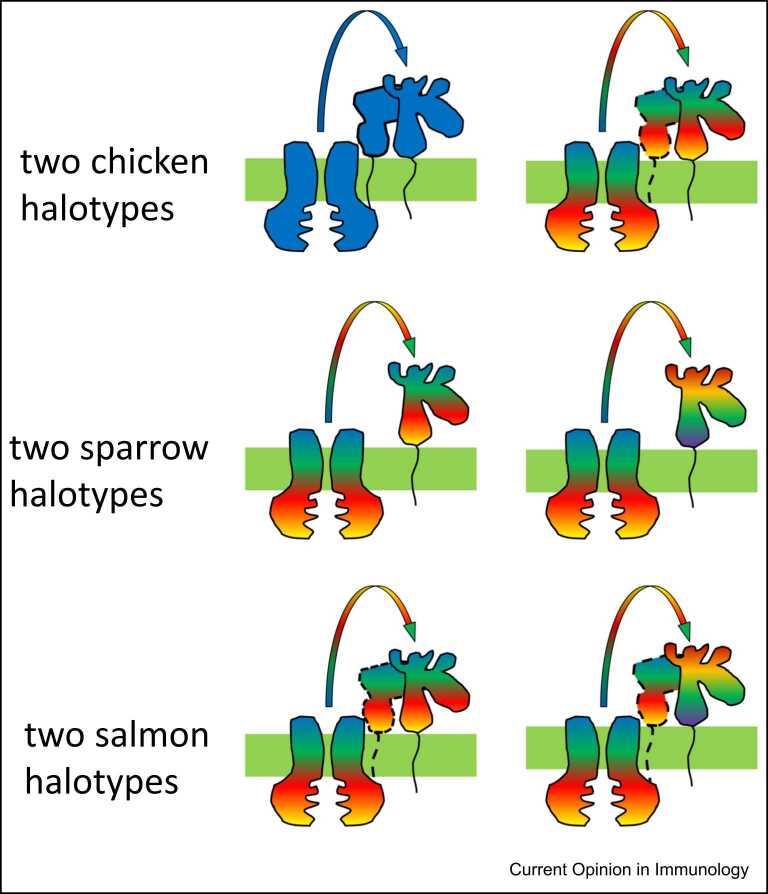
The dominantly-expressed classical class I molecules of chickens range from fastidious (solid colours) to promiscuous (rainbow colours) with TAP and tapasin genes to match, while sparrows and salmon are proposed to have only promiscuous classical class I alleles with nonpolymorphic promiscuous TAPs. Depicted are two haplotypes for chickens, one with fastidious and the other with promiscuous TAP, tapasin and class I molecules. For sparrows and salmon, the two haplotypes each have the same monomorphic promiscuous TAP molecule (with the same rainbow pattern) but different alleles of promiscuous class I molecules (with different rainbow patterns). There is no evidence for tapasin in sparrows (which would fit with the low tapasin-dependence of the proposed promiscuous class I alleles), while tapasin is found in salmon but whose role may be limited to acting only as a chaperone (as indicated by dotted lines), if the class I alleles are promiscuous as proposed.

The correlations found for the hierarchy of class I alleles can be applied to some aspects of different MHC organisations. Based on what is known for chicken BF2 and human classical class I molecules, the lack of linkage to the nonpolymorphic TAP along with the lack of a tapasin gene in sparrows may mean that alleles of the single dominantly-expressed classical class I molecule are promiscuous, getting peptides from a highly promiscuous TAP molecule and thus being tapasin-independent. Similarly, the single class I molecules of *Xenopus* frogs and the bony fish Atlantic salmon may also be promiscuous (Figure 4). The question then would become why highly promiscuous TAPs in sparrows, *Xenopus* and salmon are not supporting a multigene family of class I molecules as in mammals. One possibility is that the concept of a multigene family of similar class I molecules in mammals is not quite right, but instead the best way to imagine them is as a collection of somewhat specialised class I molecules, some promiscuous and others fastidious 5, 52•. Indeed, HLA-C is primarily an NK cell ligand, and there are indications that HLA-A and HLA-B are focused on presentation of different kinds of viruses 53, 54. In fact, one might ask why highly promiscuous TAP alleles in chickens do not support a multigene family of class I genes.

## Conclusions

Among the welter of details described in this review are many differences from what is found in textbooks based primarily on humans and mice. Of immediate interest might be the function of the third tapasin-family member outside of mammals and how the class I molecules of some birds acquire their peptides in the absence of tapasin family members, how positive selection for avian T cells occurs without inducible proteasome components, the way in which classical class II molecules of most fish acquire their peptides in absence of DM, and the way in which the fish like cod deal with pathogens in the absence of the class II pathway. Also of interest are the importance of co-evolution between APPL and classical MHC molecules given the current understanding of promiscuous and fastidious class I molecules (particularly the reasons for a single dominantly-expressed class I molecule in many NMJVs versus a multigene family as in mammals, and the polymorphism of chicken DM genes 55, 56 given the suggestion of promiscuous and fastidious class II molecules in humans [57]), the functions of the many apparently nonclassical class I and class II B genes found outside of mammals, the function of the original W molecules (speculated to be ligands of γδ T cells, [27]) as well as the current ones, and the mechanistic reasons for the appearance of the W/class I-specific interdomain contacts.

Finally, nearly everything known about the functions of the MHC has been determined by correlations using outbred humans and experiments using inbred mice. NMJV species may validate some concepts and challenge others, as has been suggested for chickens [6••]. In the longer term, any answers to these questions will lead to more questions, some of which we cannot even imagine at present.

## CRediT authorship contribution statement

RM and JK both contributed to preparation of the manuscript.

## Conflict of interest statement

The authors declare that they have no known competing financial interests or personal relationships that could have appeared to influence the work reported in this paper.

## Funding

RM was supported by 10.13039/501100000268Biotechnology and Biological Sciences Research Council (BBSRC DTP PhD in Biological Sciences) and JK was supported by an Investigator Award of the Wellcome Trust (grant number 110106/A/15/Z).

## References and recommended reading

Papers of particular interest, published within the period of review, have been highlighted as:

• of special interest

•• of outstanding interest.
